# Supplementary material for: Time-Restricted Feeding Attenuates Metabolic Dysfunction-Associated Steatohepatitis and Hepatocellular Carcinoma in Obese Male Mice
Source: Cancers (Basel). 2024 Apr 16;16(8):1513. doi: 10.3390/cancers16081513 (PMC11048121; doi:10.3390/cancers16081513)
Supplement: Supplementary file 1 [file cancers-16-01513-s001.zip › cancers-2928542-supplementary.pdf]

Supplementary Table S1: Primers for QPCR

Gene name	Oligonucleotides sequences	
**Cidea**	Forward: TGACATTCATGGGATTGCAGAC<br>Reverse: GGCCAGTTGTGATGACTAAGAC	Integrated DNA Technologies
**Cidec**	Forward: GATGGACTACGCCATGAAGTC<br>Reverse: GTGCTCACTGCCACATGC	Integrated DNA Technologies
**Cd36**	Forward: GGACATTGAGATTCTTTTCCTCTG<br>Reverse: GCAAAGGCATTGGCTGGAAGAAC	Integrated DNA Technologies
**Acc2**	Forward: TGAATCTCACGCGCCTACTA<br>Reverse: GCCTCTCTTCACCAGATGGA	Integrated DNA Technologies
**Fasn**	Forward: GGCATCATTGGGCACTCCTT<br>Reverse: GCTGCAAGCACAGCCTCTCT	Integrated DNA Technologies
**Emr1 (F4/80)**	Forward: CTTTGGCTATGGGCTTCCAGTC<br>Reverse: GCAAGGAGGACAGAGTTTATCGTG	Integrated DNA Technologies
**Clec4f**	Forward: GAGGCCGAGCTGAACAGAG<br>Reverse: TGTGAAGCCACCACAAAAAGAG	Integrated DNA Technologies
**Ccl2**	Forward: TTAATGCCCCACTCACCTGC<br>Reverse: GAGCTTGGTGACAAATACTACAGC	Integrated DNA Technologies
**Cd68**	Forward: TGTCTGATCTTGCTAGGACCG<br>Reverse: GAGAGTAACGGCCTTTTTGTGA	Integrated DNA Technologies
**IL6**	Forward: GCTACCAAACTGGATATAATCAGGA<br>Reverse: CCAGGTAGCTATGGTACTCCAGAA	Integrated DNA Technologies
**IL10**	Forward: GCTCTTACTGACTGGCATGAG<br>Reverse: CGCAGCTCTAGGAGCATGTG	Integrated DNA Technologies
**TNFα**	Forward: TGAGACCAGCCTGTGCTATG<br>Reverse: AAGCCAAAGCTGGTCAGTCTA	Integrated DNA Technologies
**Fn1**	Forward: TGGTGGCCACTAAATACGAA<br>Reverse: GGAGGGCTAACATTCTCCAG	Integrated DNA Technologies
**Timp1**	Forward: CCAGAGCCGTCACTTTGC TT<br>Reverse: AGGAAAAGTAGACAGT GTTCAGGCTT	Integrated DNA Technologies
**Acta2**	Forward:CTGAGCGTGGCTATTCCTTC<br>Reverse:CTTCTGCATCCTGTCAGCAA	Integrated DNA Technologies
**Col1a1**	Forward:CACCCTCAAGAGCCTGAGTC<br>Reverse:GTTCGGGCTGATGTACCAGT	Integrated DNA Technologies
**Col13a1**	Forward: GACCAAAAGGTGATGCTGGACAG<br>Reverse:CAAGACCTCGTGCTCCAGTTAG	Integrated DNA Technologies
**Col14a1**	Forward:ATGGCTTGCCTGGAGAGATAGG<br>Reverse:TGGTTGCCCTTTGAGTCCTGGA	Integrated DNA Technologies
